# CircPVT1 promotes proliferation of lung squamous cell carcinoma by binding to miR-30d/e

**DOI:** 10.1186/s13046-021-01976-w

**Published:** 2021-06-10

**Authors:** Jie Shi, Xin Lv, Lizhong Zeng, Wei Li, Yujie Zhong, Jingyan Yuan, Shanshan Deng, Boxuan Liu, Bo Yuan, Yang Chen, Zongjuan Ming, Xia Yang, Ping Fang, Shuanying Yang, Guoan Chen

**Affiliations:** 1 Department of Pulmonary and Critical Care Medicine, The Second Affiliated Hospital of Xi’an Jiaotong University, 157th Xiwu Road, Xi’an, 710000 People’s Republic of China; 2 School of Medicine, Southern University of Science and Technology, 1088th Xueyuan Road, Shenzhen, 518055 People’s Republic of China

**Keywords:** circPVT1, Lung squamous cell carcinoma, HuR, miR-30, CCNF

## Abstract

**Background:**

Circular RNAs (circRNAs) are a new type of extensive non-coding RNAs that regulate the activation and progression of different human diseases, including cancer. However, information on the underlying mechanisms and clinical significance of circRNAs in lung squamous cell carcinoma (LUSC) remains scant.

**Methods:**

The expression profile of RNAs in 8 LUSC tissues, and 9 healthy lung tissues were assayed using RNA sequencing (RNA-seq) techniques. Real-time quantitative polymerase chain reaction (qRT-PCR) was used to profile the expression of circPVT1 and its relationship with the prognosis of LUSC, i.e., survival analysis. Moreover, in vitro and in vivo experiments were performed to evaluate the impacts of circPVT1 on the growth of tumors. RNA pull-down tests, mass spectrometry, dual-luciferase reporter assessment, and RNA immune-precipitation tests were further conducted to interrogate the cross-talk between circPVT1, HuR, or miR-30d/e in LUSC.

**Results:**

Our data showed that circPVT1 was upregulated in LUSC tissues, serum, and cell lines. LUSC patients with higher circPVT1 expression exhibited shorter survival rates. The in vivo *and* in vitro data revealed that circPVT1 promotes the proliferation of LUSC cells. Additionally, mechanistic analysis showed that HuR regulated circPVT1. On the other hand, circPVT1 acted as a competing endogenous RNA (ceRNA) of miR-30d and miR-30e in alleviating the suppressive influences of miR-30d and miR-30e on its target cyclin F (CCNF).

**Conclusion:**

CircPVT1 promotes LUSC progression via HuR/circPVT1/miR-30d and miR-30e/CCNF cascade. Also, it acts as a novel diagnostic biomarker or treatment target of individuals diagnosed with LUSC.

**Supplementary Information:**

The online version contains supplementary material available at 10.1186/s13046-021-01976-w.

## Background

Lung cancer (LC) accounts for the highest rate of cancer incidence and mortalities across the globe. Notably, 2 types of LCs have been reported, including non-small cell lung cancer (NSCLC) and small cell lung cancer (SCLC), accounting for up to 85, and 15% of all cases, respectively [1]. NSCLC patients present with dismal clinical outcomes, where, only 15% survive for 5 years or more [2]. The most prevalent types of NSCLC include lung adenocarcinoma (LUAD) and lung squamous cell carcinoma (LUSC), which accounts for approximately 90% of all cases. Moreover, LUSC has been associated with annual fatalities of more than 400, 000 [3]. Unlike LUAD, the spectrum of targeted therapies achievable for LUSC is limited. To enhance the prognosis of LUSC, there is a desperate and urgent need for a profound understanding of the molecular mechanisms of LUSC and the development of individualized treatment strategies.

Circular RNAs (circRNAs) are a new type of extensive non-coding RNAs, regulating pivotal functions in distinct kinds of cancers. They generate non-polar or non- polyadenylated tailed covalently closed continuous looped structures, a phenomenon that enhances their stability and abundance compared to their well-established linear coding RNAs of similar cellular genes [4, 5]. In addition to tissues, circRNAs have been documented in various body fluids, including urine, saliva, and blood, indicating that they can be applied as non-invasive circulating diagnostic biomarkers for cancer. Previous reports showed that circRNAs modulate the expression of genes during transcription, post-transcription, and translation. The circRNAs in cytoplasm possess microRNA (miRNA) response elements (MREs), which competitively combined with miRNAs, downregulating their expression, reducing their function, hence promoting expressions of miRNAs targeted genes. This implies that circRNAs are linked to diverse biological processes promoting the progression of cancer, i.e., cell proliferation, migration, infiltration, and apoptosis.

Herein, we constructed RNA expression data from LUSC tissues, then established numerous candidates of circRNAs (at least 2 unique back spliced reads). Subsequently, we characterized one circRNA derived from the PVT1 locus (circPVT1), which is often up-regulated in LUSC individuals. Functional assessments revealed that HuR regulated the expression level of circPVT1 and circPVT1 improved cell proliferation via sponging miR-30d/e members. Moreover, circPVT1 was documented as an independent prognostic biomarker for survival in individuals diagnosed with LUSC.

## Methods

### Human samples

For RNA-seq analysis, total RNAs from 8 LUSC tissues and 9 healthy lung tissues, including 8 matched vicinal healthy tissues and 1 other healthy lung tissue, were acquired from the subjects. Cancer samples and healthy tissues were collected from individuals diagnosed with LUSC admitted to the second affiliated hospital of Xi’an Jiaotong University (Xi’an China). All the participants recruited had not undergone previous surgery, radiotherapy, chemotherapy, targeted or immunotherapy and the tissues were examined histologically. Clinically pathological features comprising age, differentiation grade, gender, site of the tumor, TNM stage and tumor size are shown in Table 1. The patients were followed-up between the time of surgery and disease progression date, demise, or the last clinical assessment. All the patients provided informed consent. Besides, this study was approved by the Clinical Research Ethics Committee of the second affiliated hospital of Xi’an Jiaotong University.

**Table 1:** Correlation between circPVT1 expression and clinicopathological characteristics of LUSC patients (*N* = 104)

Characteristics	Total number	Low expression (*N* = 50)	High expression (*N* = 54)	*P* value
Age				0.3216
< 60	44	24	20	
≥ 60	60	26	34	
Gender				0.1936
Male	74	39	35	
Female	30	11	19	
Smoking history				0.1733
Yes	78	41	37	
No	26	9	17	
TNM stage				0.0001
I-II	50	35	15	
III-IV	54	16	38	
Tumor size				0.0468
≤ 5 cm	63	36	27	
> 5 cm	41	15	26	
Lymph node metastasis				0.0014
Yes	60	21	39	
No	44	30	14	
Differentiation				
Not poor	48	28	20	0.1151
Poor	56	23	33	

### RNA-seq analysis

For the generation of the RNA library, processing of the total RNA (tRNA) was conducted using the TruSeq Stranded Total RNA with Ribo-Zero Gold (Illumina, USA). Following the removal of rRNA, preparation of the strand-distinct RNA-seq libraries was conducted based on the manufacturer’s instructions. Briefly, fragmentation of the ribosome free RNA was conducted. Thereafter, 1st strand cDNA was generated from the fragmented RNA, followed by the preparation of the 2nd strand cDNA using the random hexamers, and dUTP, respectively. Subsequently, end repairing of the generated cDNA fragments was conducted, followed by poly-A tailing, and eventually ligation with indexed adapters. Purification of these ligated cDNA libraries was performed, then uracil DNA glycosylase treatment was applied to degrade the 2nd strand cDNA. Library enrichment of the 1st strand purified cDNA products was performed via 13–16 amplification cycles in PCR. Library quality was evaluated using a Bioanalyzer 2200 (Agilent, Santa Clara, CA), then sequencing was conducted on a HiSeq X machine (Illumina, San Diego, CA) via a run of 150 bp pair-ends.

### Cell culture and treatments

The A549, H520, H226, SKMES-1, and H1270 cells were obtained from American Type Culture Collection (ATCC). Then, the cells were incubated in RPMI1640, enriched with fetal bovine serum (FBS) (10%) at 37 °C and 5% CO_2_. Blocking of transcription was accomplished by the introduction of DMSO (2 mg/ml) (Sigma-Aldrich, St. Louis, MO, USA), which was the control of the cell growth medium.

### RNA purification and qRT–PCR

The TRIzol reagent (Life Technologies, Carlsbad, CA) was used in the purification of tRNA from the complete-cell lysates. Afterward, quantification of the mature miRNA was conducted via the TaqMan MicroRNA assays (Life Technologies) as an internal standard. On the other hand, cDNA generation from the 500 ng tRNA was performed using the PrimeScript RT Master Mix kit (Takara, Dalian, China) to quantify the mRNA and circRNA. qRT-PCR tests were conducted using the SYBR Premix Ex Taq II (Takara).

Divergent primers complementary to the circRNA distal ends were adopted in the quantification of the circRNA. The absolute RNA quantity was determined, the pure PCR products generated from amplification of cDNA complementary to the circPVT1 and purified, were serially diluted to form a standard curve.

### Vector construction

The full-length sequence of circPVT1 was synthesized by chemical synthesis, then the sequence was constructed into the GV486 vector (Genechem Co.,Ltd., Shanghai) using the two restriction sites of KpnI / BamHI. There are two intron sequences on both sides of the KpnI / BamHI digestion site, which can promote the formation of circRNA between the two intron sequences. The full-length sequence of HuR and CCNF was synthesized, then the sequence was constructed into the PCDNA3.1vector (Jinkairui, Wuhan, China).

### Oligonucleotide transfection

RiboBio (Guangzhou, China) synthesized the siRNA (Additional file 1), miRNA mimics, and miRNA inhibitors. Transfection of the cells was conducted using the Lipofectamine RNAiMax generated from Life Technologies.

### CCK-8 evaluation

The H520, H2170 cell proliferation was assessed using the CCK-8 kit (Doindo, Japan). Triplicates of 3.5 × 10^3^ transfected cells were incubated in 100 ml in 96-well dishes. At 0 h, 24 h, 48 h and 72 h, 10 ml of the CCK-8 reagent was added to every well, followed by 2 h incubation at 37 °C. The automatic microplate reader (Synergy4; BioTek, Winooski, VT, USA) was set at 450 nm to acquire the optical density of each well.

### EdU test

The cell-light EdU DNA Cell Proliferation Kit (obtained from RiboBio, Shanghai, China) was used to inspect the 5-ethynyl-20-deoxyuridine (EdU). H520 or H2170 (1 × 10^4^) cells were each planted in 96-well dishes for insertion with si-circPVT1, vector-circPVT1, mock or negative control (NC) oligonucleotide via transfection. After incubation for 48 h at 5% CO_2_ and 37 °C, 50 mM EdU was added to the cells followed by another 2 h of incubation. The cells were fixed using paraformaldehyde (4%), stained using the Apollo Dye Solution for cell proliferation, whereas DAPI staining was performed for nucleic acids. The rate of cell proliferation was computed as outlined in manufacturer provided instructions. The images were visualized under a fluorescence microscope (Olympus FSX100).

### Luciferase reporter assay

CircPVT1, miR-30d, and miR-30e sequences and their correspondent mutant forms without docking sites were constructed and sub-cloned into luciferase enzyme reporter vector, termed circPVT1-WT, circPVT1-Mut, miR-30d-WT, miR-30d –Mut, miR-30e-WT, miR-30e –Mut. Sequencing was applied to verify these plasmids. Moreover, the relative luciferase enzyme activity was inspected using the Dual-Luciferase Assay Kit (obtained from Promega, Madison, WI, USA) following the protocol provided by the manufacturer.

### Colony-forming assessment

Further, 1 × 10^3^ cells were plated onto 60 mm culture plates. Then, the cells were incubated at 37 °C and 5% CO_2_ for 10 days. Subsequently, colony staining was performed using crystal violet for 30 min (Sigma-Aldrich) and the cells were enumerated.

### Western blot analysis

Cell homogenization was performed on ice using the lysis constituting Hepes (50 mM, pH 7.5), protease inhibitor cocktail (Roche), EDTA (5 mM, pH 8.0), NaCl (150 mM), β-glycerophosphate (20 mM), NP40 (0.5%), sodium orthovanadate (0.1 mM), dithiothreitol (DTT) (1 mM), NaF (50 mM), PMSF (1 mM) and MLUADl2 (10 mM). Clarification of the cell lysates was performed via 10 min centrifugation at 4 °C. Thereafter, 30μg/lane of the proteins were loaded into wells and fractionated using SDS-PAGE gels (10%) and transfer-embedded onto nitrocellulose membranes. Conjugation of the immunoblots with primary rabbit polyclonal anti-CCNF (Abcam, USA), and mouse monoclonal anti-GAPDH (Calbiochem) was conducted. The immunostained bands were developed, and the inspected via a chemiluminescent strategy.

### RNA immunoprecipitation (RIP) evaluation

The Magna RIP kit (Millipore, Billerica, MA, USA) was used in the RIP assay following the manufacturer’s instructions. H520 cells were lysed using the complete RNA lysis buffer. Then, the cell lysates with magnetic beads probed anti-HuR (Abcam, USA) or negative control IgG antibody (Abcam, USA) were incubated at 4 °C for 4 h. The beads were rinsed using the washing buffer. Eventually, immunoprecipitated RNA was purified, followed by enrichment to evaluate the targeted RNAs via qRT-PCR.

### RNA pull-down

CircPVT1 probe (5′-GCCAAAAGATCAGGCCTCAAGCCCAGCTGAGCGCCG.

GATGGAACG-3′-Biotin), and the control probe were synthesized (Jinkairui, Wuhan, China). H520 cells were lysed using the lysis buffer. Subsequently, the lysed cells were incubated with the respective circPVT1 probes. Then, the cell lysates were incubated with the streptavidin-coated magnetic beads to pull down the biotin-conjugated RNA complex. The beads were rinsed, and the RNA complex was purified using TRIzol (Takara, Dalian, China). Lastly, the immunoprecipitated RNA and protein were analyzed by miRNA sequencing and proteomic identification (LC-MS/MS).

### Animal experiments

The Xi’an Jiaotong University Animal Care and Use Committee approved all the mice experiments. Besides, the mice experiment protocols adhered to the guidelines of the National Institutes of Health. Stably down expressed cell lines were established through transfection of H520 cells and selection using puromycin. H520cells were infected using the lentiviruses (Jikai, Shanghai, China) comprising NC and si-cPVT1. Regarding the xenograft assays, diverse types of H520 cells (2 × 10^6^) were subcutaneously administered into nude mice. The tumor volume and body weight were inspected once every week. The mice were sacrificed after 7 weeks, then the tumors were harvested for subsequent experimentation.

### Immunohistochemistry (IHC)

For the IHC assay, the paraffin-embedded segments were probed overnight at 4 °C with the primary antibodies against CCNF (1:100) (Abcam, CA). Then, the secondary antibodies were conjugated via incubation at 37 °C for 1 h. The HRP-conjugated streptavidin solution was incubated with the segments for 10 min, followed by staining using DAB.

### Statistical analysis

Statistical analyses were performed using GraphPad Prism 8. Data were indicated as mean ± standard deviation (SD). The student’s t-test, or χ^2^ test was used to evaluate the difference between study groups. Moreover, the Kaplan-Meier approach was applied to establish the survival rates. Furthermore, the relationship linking study groups was determined through the Pearson correlation. Notably, *P* < 0.05 denoted statistical significance.

## Results

### Differential circRNA expression in LUSC

To elucidate the expression patterns of circRNA and mRNA in LUSC, RNA-seq tools were used to sequence RNA from LUSC and normal lung tissues. Then, the DE-Seq algorithm was applied in filtering the differentially expressed genes. Besides, significant differences in expression were determined by *P*-value and FDR evaluation as follows: Fold Change > 2 or < 0.5, *P*-value < 0.05, FDR < 0.05.

**Fig. 1:**
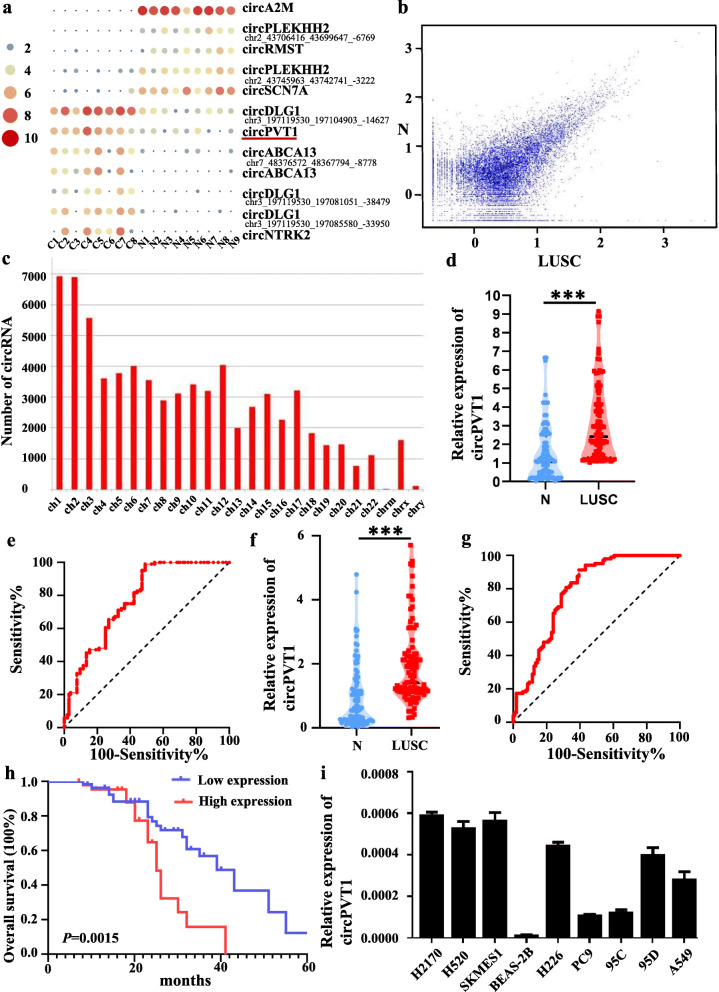
Expression profiles of circRNA in LUSC. **a** The cluster heat maps displayed an increased and decreased circRNAs. Each column indicates a sample while each row indicates an individual circRNA. The red and blue strips represent high and low expression, respectively; **b** Scatter plot of circRNAs. **c** The distribution of differentially expressed circRNAs on chromosomes between LUSC tissues and non-tumor tissues; **d** Relative expression level of circPVT1 in LUSC tissues (T) and adjacent normal tissues (N) (*n* = 104) using qRT-PCR. CircPVT1 expression was significantly higher in LUSC tissues, compared to that in adjacent normal tissues; **e** Diagnostic efficacy of circPVT1 in LUSC tissue was detected via ROC curve analysis; **f** Relative expression level of circPVT1 in LUSC tissues (T) and adjacent normal serum (N) (*n* = 110) using qRT-PCR. CircPVT1 expression was significantly higher in LUSC serum, compared to that in adjacent normal tissues; **g** Diagnostic efficacy of circPVT1 in LUSC serum was detected via ROC curve analysis; **h** Kaplan-Meier analysis of overall survival curves based on circPVT1expression levels in LUSC patients; **i** Relative expression level of in lung cancer cell lines and BEAS-2B cell using qRT-PCR. *P < 0.05 versus BEAS-2B cells. Data were showed as mean ± SD, *P < 0.05, ***P < 0.001

Relative to the normal tissues, the expression analysis revealed that the circRNA transcripts were differentially expressed in cancerous tissues (Fig. 1a, b and c). Among the 12 differentially expressed circRNAs, 7 were upmodulated while 5 were downmodulated in LUSC tissues in contrast with the normal tissues. Among the 7 up-modulated circRNA, circPVT1 demonstrated the most significant effect of cell proliferation, hence, circPVT1 was selected as a research target.

### Upregulation of CircPVT1 in LUSC samples correlates with the prognosis of LUSC patients

To establish whether the expression of circPVT1 was changed in LUSC, the expression of circPVT1 was assessed in 104 pairs of LUSC tissues and 110 pairs of LUSC serum using qRT-PCR. In contrast with the vicinal normal tissues, our data showed upregulated circPVT1 expressions in LUSC tissues (Fig. 1d) and serum (Fig. 1f). Similarly, the levels of circPVT1 expressions in lung cancer cell lines (H2170, H520, SKMES1, H226, 95c, 95d, A549, and PC9) were upregulated in contrast with the healthy human bronchial epithelial cell line (BEAS-2B) (Fig. 1i).

Additionally, the diagnostic significance of the expression of circPVT1 in tissues and serum of LUSC patients were evaluated. Consequently, for the circPVT1 expression in tissues, the ROC analysis exhibited an area under curve (AUC) of 0.774, and a diagnostic sensitivity and specificity of 97.1 and 51% (95% CI = 0.711–0.837), respectively (Fig. 1e). For serum circPVT1 expressions, the AUC was 0.789, and the diagnostic sensitivity and specificity were 91.3 and 60.6% (95% CI = 0.726–0.852), respectively (Fig. 1g). This indicated that serum circPVT1 potentially acts as a biomarker for the diagnosis of LUSC. To investigate the relationship between circPVT1 and patient prognosis, a cohort of 104 LUSC subjects with clinicopathological features and survival data was analyzed. As a result, the content of circPVT1 expression was directly linked to tumor node metastasis stage (TNM) (*P* = .0001), lymph node metastasis (*P* = .0014) and tumor size ((*P* = .0468), but not with other clinicopathological characteristics including age, or gender (Table 1). Multivariate analysis identified circPVT1 expression as an independent predictor of OS (Table 2). Besides, LUSC subjects with lower circPVT1 expression exhibited a better overall survival relative to those with elevated circPVT1 expression (Fig. 1h). Collectively, these data indicate that circPVT1 was overexpressed in LUSC tissues and serum, also, higher circPVT1 expression caused a poor prognosis of LUSC patients.

**Table 2:** Univariate and Multivariate Analyses of Factors Associated with Overall Survival

Facrors	OS			
	Univariate	Multivariate		
	*p*	HR	95%CI	*P* value
Sex (Female vs. Male)	0.841			NA
Age (years)(< 60 vs. ≥60)	0.045			NA
Smoking history (Yes vs. No)	0.518			NA
TNM stage(I-II vs. III-IV)	0.002	1.940	0.798–4.713	O.143
Tumor size(≤5 cm vs. > 5 cm)	0.000	1.927	0.882–4.207	0.100
Lymph node metastasis (Yes vs. No)	0.007	I.097	0.499–2.410	0.819
Differentiation (Not poor vs. Poor)	0.001	1.367	0.759–2.462	0.297
CircPVT1 expression (High vs. Low)	0.000	2.019	1.118–3.644	0.020

*OS* Overall survival, *NA* Not adopted, *95%CI* 95% confidence interval, *HR* Hazard ratio; Cox proportional hazards regression model

### CircPVT1 promotes cell proliferation in vitro

**Fig. 2:**
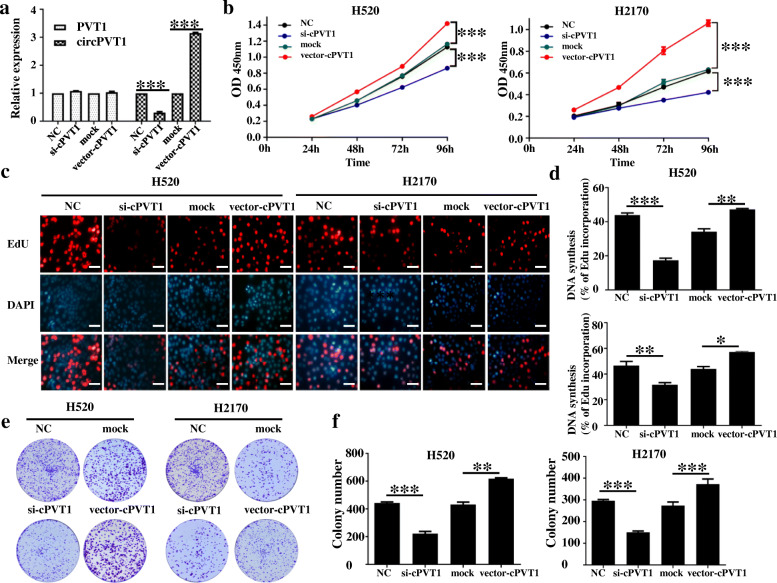
CircPVT1 promotes LUSC cell proliferation. **a** qRT-PCR analysis of circPVT1 and PVT1 RNA expression in H520 cells after treatment with circPVT1 expression vector, mock, si-cPVT1 or NC; **b** Assessment of proliferation in H520 and H2170 cells transfected with circPVT1 expression vector, mock, si-cPVT1 or NC by CCK-8 assay; **c** and **d** Assessment of DNA synthesis using an EdU assay in H520 and H2170 cells transfected with circPVT1 expression vector, mock, si-cPVT1 or NC. Micrographs represent at least 3 experiments. Scale bar = 200 μm. **e** and **f**. Colony formation assay in H520 and H2170 cells transfected with circPVT1 expression vector, mock, si-cPVT1, or NC. Data were showed as mean ± SD, *P < 0.05; **P < 0.01; ***P < 0.001

Further, the functional role of circPVT1 in LUSC progression was investigated. First, the expression of circPVT1 in H520 and H2170 cell lines were silenced by transfecting with circPVT1 siRNA. To evaluate the role of circPVT1 in LUSC cells, 2 siRNAs were designed against circPVT1 and used to silence circPVT1 without influencing PVT1 level. The circular transcript expression vector circPVT1 was effectively constructed, as it could upregulate circPVT1 expression level rather than mRNA (Fig. 2a). CCK-8 (Fig. 2b) and EdU (Fig. 2c, d) assays data illustrated that si-circPVT1 could inhibited cell proliferation in H520 or H2170 cell lines, whereas over-expression of circPVT1 might promoted cell proliferation in H520 or H2170 cells lines. Further, colony formation evaluations revealed significantly improved cell cloning potentials of H520 or H2170 cells. This improvement was related to the upregulation of circPVT1 but was significantly impaired by the down-modulation of circPVT1 (Fig. 2e, f). These data suggested that circPVT1 promotes LUSC cell proliferation.

### CircPVT1 is actively triggered by HuR in LUSC cells

**Fig. 3:**
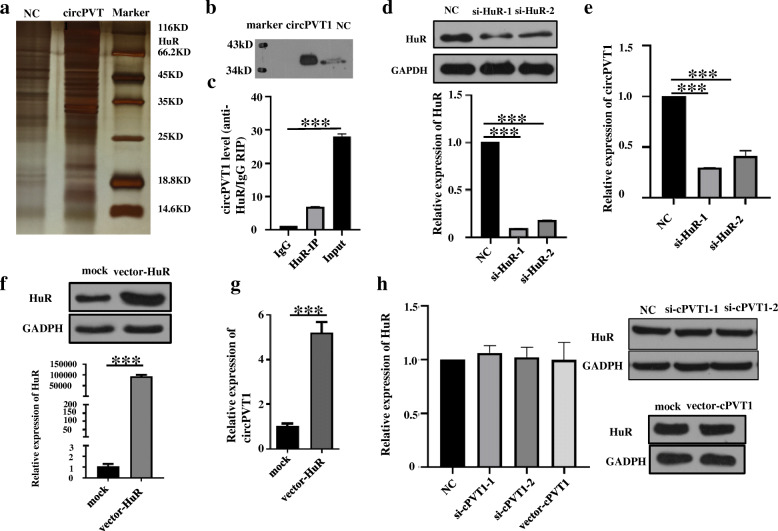
CircPVT1 is actively induced by HuR. **a** Silver staining of biotinylated circPVT1-associated proteins; **b** Western blotting of HuR from circPVT1 pull-down assays; **c** RIP confirmed the relationship between circPVT1 and HuR in H520 cells. GAPDH mRNA was used as a non-HuR target control; **d** The mRNA and protein level of HuR were significantly reduced by a HuR-siRNA as determined by qRT-PCR (below) and western blot (up); **e** CircPVT1was markedly reduced by HuR-siRNA as established by qRT-PCR; **f** The mRNA and protein level of HuR was markedly elevated by a vector-HuR as determined by qRT-PCR (below) and western blot (up); **g** CircPVT1was markedly upregulated by vector-HuR as determined by qRT-PCR; **h** The mRNA and protein level of HuR were not changed by circPVT1siRNA or circPVT1 expression vector determined by qRT-PCR (left) and western blot (right). Data are shown as mean ± SD, **P < 0.01;***P < 0.001

Mounting research evidence confirmed that multiple key proteins bind to circRNAs and mediate RNA dysregulation in human cancer cells. To search for proteins interacting with the circPVT1, RNA pull-down experiments were conducted. Mass spectrometry discovered 112 proteins associated with circPVT1, including HuR (Fig. 3a, b). HuR binds and stabilize cancer-associated mRNAs. However, whether HuR affects circPVT1 remains unclarified. To confirm the potential interaction between HUR and circPVT1, HuR specific antibody was used to perform RNA precipitation. Through RIP and qRT–PCR, a direct interaction was found between HuR and circPVT1 (Fig. 3c). Further, 2 siRNAs were designed against HuR (Fig. 3d) and downregulation of HuR suppressed the expression of circPVT1 (Fig. 3e). The vector HuR was effectively constructed, as it could upregulate HuR expression level rather than mRNA (Fig. 3f) and HuR overexpression upregulated the expression of circPVT1 (Fig. 3g). Whereas, circPVT1 did not change the level of HuR (Fig. 3h). Therefore, it was inferred that the up-modulation of circPVT1 in LUSC might have been triggered by HuR.

### CircPVT1 sponges the miR-30d/e

**Fig. 4:**
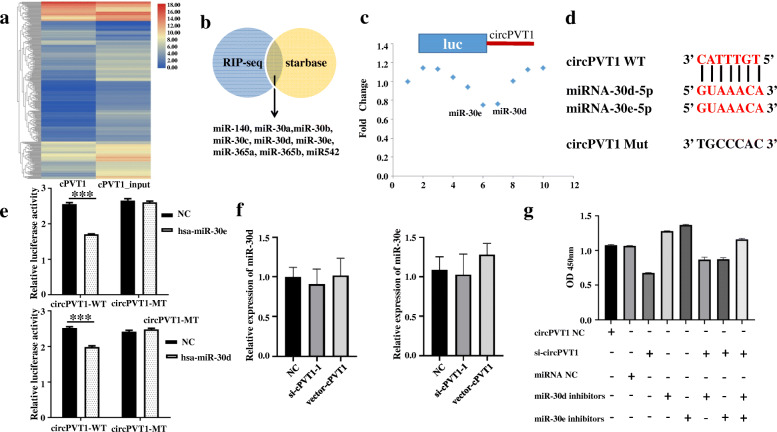
CirPVT1 binds to miR-30d and miR-30e. **a** The cluster heat maps displayed the miRNAs binding to circPVT1; **b** Screened miRNA that possibly binds to circPVT1; **c** A luciferase reporter assay was used to detect the luciferase activity of LUC-cPVT1 in H520 cells transfected with miRNA mimics to identify miRNAs that bind to the circPVT1 sequence; **d** A schematic drawing shows the putative binding sites of miR-30d and miR-30e for circPVT1; **e** A luciferase reporter assay was used to detect the luciferase activity of LUC-cPVT1 or the LUC-cPVT1-mutant in H520 cells co-transfected with miRNA 30d mimics (down) and miRNA 30e mimics (up); **f** The relative level of miR-30d and miR-30e was measured by RT-qPCR in the H520 cells transfected with circPVT1 expression vector, mock, si-cPVT1 or NC; **g** Assessment of proliferation in H520 cells after 72 h transfected with circPVT1 siRNA or miR-30d inhibitors or miR-30d inhibitors by CCK-8 assay. Data are shown as mean ± SD, **P < 0.01;***P < 0.001

Given that circRNA has been documented as a miRNA sponge and circPVT1 is stably expressed in the cytoplasm, the plausibility of circPVT1 binding to the miRNAs was investigated. To identify the functional miRNAs potentially interacting with circPVT1 in LUSC cells, circPVT1 specific probe was used to perform RIP as described previously [6]. Through RIP and RNA-seq (Fig. 4a), 244 downregulated RNA and 66 upregulated RNA were identified. Then, the potential miRNAs were screened which were predicted by starBase 3.0.

Among the candidate miRNAs predicted by starBase 3.0 and RNA-seq, 9 miRNAs were analyzed, comprising miR-140, miR-30a, miR542, miR-30b, miR-365a, miR-30c, miR-30e, miR-365b, and miR-30d (Fig. 4b). Then, a luciferase enzyme assay was conducted. A mimic of each miRNA was co-inserted with the luciferase reporters into H520 cells via co-transfection. As expected, relative to the control, miR-30d and miR-30e reduced the luciferase reporter activity in both H520 cells (Fig. 4c) and T293 cells (FigS1). MiR-30d and miR-30e belong to the miR-30 family and share similar miRNA seed sequences. Besides, both of these miRNAs have been documented as tumor repressors [7, 8]. Since only one potential docking site of the miR-30d/e was identified within the circPVT1 sequence, the possible circPVT1 target site was mutated with the inclusion of each miRNA sequence in the 3’UTR (Fig. 4d). We established that the transfection of H520 cells with miR-30d and miR-30e had an apparent distinct influence on luciferase enzyme activity when the correspondent target regions were mutated from the luciferase reporter (Fig. 4e). qRT-PCR analysis revealed that miR-30d or miR-30e did not show significant changes after circPVT1 was overexpressed or silenced (Fig. 4f) and miR-30d or miR-30e did not decrease the level of circPVT1 (Additional file 3, FigS2).

Functionally, CCK8 assay was used to verify whether miR30d/e could reverse the function of circPVT1 on cell proliferation. As showing in Fig. 4g, downregulation of circPVT1 inhibited cell proliferation, treatment with miR-30d and 30e inhibitors promoted cell proliferation, a combination of si-cPVT1 with miR-30d or miR-30e inhibitors partially reduced the effect of miR-30d/e inhibitors to promote cell proliferation and si-cPVT1 to inhibit cell proliferation. Si-cPVT1 combined with miR-30d inhibitors and miR-30e inhibitors significantly reduced the ability of miR30d or miR-30e inhibitors to promote cell proliferation and si-cPVT1to inhibit cell proliferation. The result revealed that downregulation of circPVT1 significantly suppressed the proliferation of H520cells, and the suppression could be blocked by miR-30d or miR-30e. Our results confirmed the hypothesis that circPVT1 regulates cell proliferation by combining with miR-30d and miR-30e.

### CircPVT1 suppresses the proliferation of LUSC by sponging the miR-30d/e to regulate CCNF

**Fig. 5:**
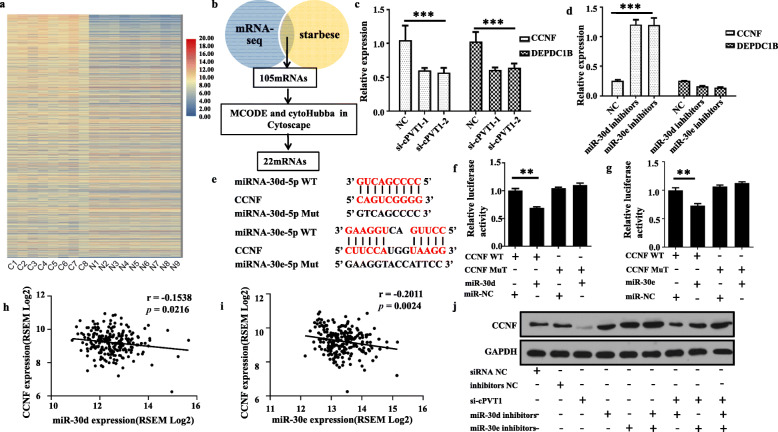
CCNF is a direct target of miR-30d and miR-30e in LUSC cells. **a** The cluster heat maps displayed the upregulated and downregulated mRNAs. Each column indicates a sample while each row indicates an individual mRNA. The red and blue strips represent high and low expression, respectively; **b** Screened mRNA that possibly binds to miR-30d and miR-30e simultaneously; **c** The mRNA level of CCNF and DEPDC1B were significantly reduced by a circPVT1siRNA as determined by qRT-PCR; **d** The mRNA level of CCNF were markedly increased by miR-30d inhibitors and miR-30d inhibitors as determined by qRT-PCR; **e** A schematic drawing shows the putative binding sites of miR-30d and miR-30e with respect to CCNF; **f** A luciferase reporter assay was used to detect the luciferase activity of LUC-miR-30d or the LUC-miR-30d-mutant in H520 cells co-transfected with CCNF; **g** A luciferase reporter assay was used to detect the luciferase activity of LUC-miR-30e or the LUC-miR-30e-mutant in H520 cells co-transfected with CCNF; **h** The correlation between CCNF and miR-30d in LUSC was evaluated in TCGA database; **i** The correlation between CCNF and miR-30e in LUSC was evaluated in TCGA database. **j** Immunoblot analysis of CCNF protein expression after co-transfection with circPVT1 siRNA or miR-30d/30e inhibitors. Data are shown as mean ± SD, **P < 0.01;, ***P < 0.001

The mRNA expression analysis of RNA-seq for elucidating the expression patterns of mRNA in LUSC revealed that 2441 mRNAs were differentially expressed. Out of the 2441 mRNAs, 835 were upregulated while 1606 mRNAs were down-regulated (Fig. 5a). To investigate whether circPVT1 exerts its effect by sponging the activity of the miR-30d/e, both Starbase 3.0 interaction prediction maps and upregulation shifts from RNA-seq were used to draw 105 RNAs based on the RNA binding with miR-30d and miR-30e predicted by starBase 3.0, upregulated RNA in LUSC. Protein-protein interaction (PPI) network was constructed using the STRING repository via the Cytoscape software. Consequently, a remarkable module with 16 nodes was established via the MCODE plugin. In total, 22 key genes were ranked by combining 6 methods as highlighted by cytoHubba (Fig. 5b). Out of the 20 genes (Additional file 4, FigS3), cyclin F (CCNF) and DEP domain-containing protein 1B (DEPDC1B) were consistently down-regulated after inhibition circPVT1 expression (Fig. 5c). Using qRT-PCR, the expression of these 2 genes after conditional knockdown of miR-30d or miR-30e was evaluated. Our results revealed that silencing of miR-30d or miR-30e improves the expression of CCNF in H520 cells (Fig. 5d).

Further, the luciferase reporter assays were used to evaluate whether miR-30d or miR-30e directly binds to CCNF 3′ UTR in H520 cells (Fig. 5e). The findings illustrated that overexpression of miR-30d or miR-30e reduced the luciferase activity of a wild-type 3’UTR of CCNF reporter, and not the mutant one (Fig. 5f, g). Using the cancer genome atlas (TCGA) repository, CCNF was inversely linked to the expression of miR-30d (Fig. 5h) or miR-30e (Fig. 5i) in LUSC. On the other hand, western blot illustrated that down-expression of circ-PVT1 significantly repressed the expression of CCNF, and the suppression could be blocked by miR-30d or (and) miR-30e (Fig. 5j). Collectively, these findings suggested that circPVT1 regulates CCNF via the sponge activity of miR-30d and miR-30e in LUSC.

### CCNF promotes cell proliferation in vitro

**Fig. 6:**
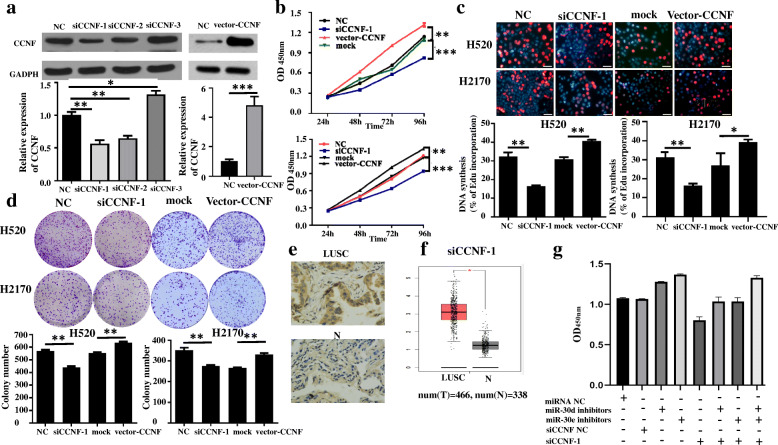
CCNF promotes LUSC cell proliferation. **a** qRT-PCR and western blot analysis of CCNF expression in H520 cells after treatment with si-CCNF, NC, CCNF vector or mock; **b** Assessment of proliferation in H520 and H2170 cells transfected with si-CCNF, NC, CCNF vector or mock by CCK-8 assay; **c** Assessment of DNA synthesis using an EdU assay in H520 and H2170 cells transfected with s si-CCNF, NC, CCNF vector or mock. Scale bar = 200 μm; **d** Colony formation assay in H520 and H2170 cells transfected with si-CCNF, NC, CCNF vector or mock; **e** IHC analysis of expression levels of CCNF between LUSC tissues and matched adjacent non-tumor tissues.; **f** Relative expression of CCNF in LUSC tissues compared to normal tissues analyzed using TCGA data; **g** Assessment of proliferation in H520 cells after 72 h transfected with CCNF siRNA or miR-30d inhibitors or miR-30d inhibitors by CCK-8 assay. Data are shown as mean ± SD, *P < 0.05; **P < 0.01; ***P < 0.001

So far, the role of CNNF in NSCLS remains unreported, as such, this work further investigated the functional role of CCNF in LUSC progression. Three siRNAs against CCNF and 1 vector CCNF were effectively constructed and their knockdown and overexpression efficiencies were verified by qRT–PCR and western blot (Fig. 6a). Data on CCK-8(Fig. 6b and FigS4) and EdU assays (Fig. 6c) illustrated that siCCNF inhibited cell proliferation, whereas over-expression of CCNF might promoted cell proliferation in H520 or H2170 cells lines. Further, colony formation evaluations illustrated that the cell cloning potentials of H520 or H2170 cells were significantly impaired by down-modulation of CCNF and was upregulated by the upregulation of CCNF (Fig. 6d). IHC findings revealed that CCNF was remarkably up modulated in LUSC tissues (Fig. 6e). This was also confirmed TCGA repository (Fig. 6f). Functionally, CCK8 assay revealed that downregulation of miR-30d and miR-30e remarkably promoted the proliferation of H520 cells, a promotion which could be blocked by CCNF (Fig. 6g). Our findings verified that CCNF promotes LUSC cell proliferation and partly reverses the effect of promoting cell proliferation of miR30d and miR30e.

### CircPVT1 suppresses growth of xenograft tumor in vivo

**Fig. 7:**
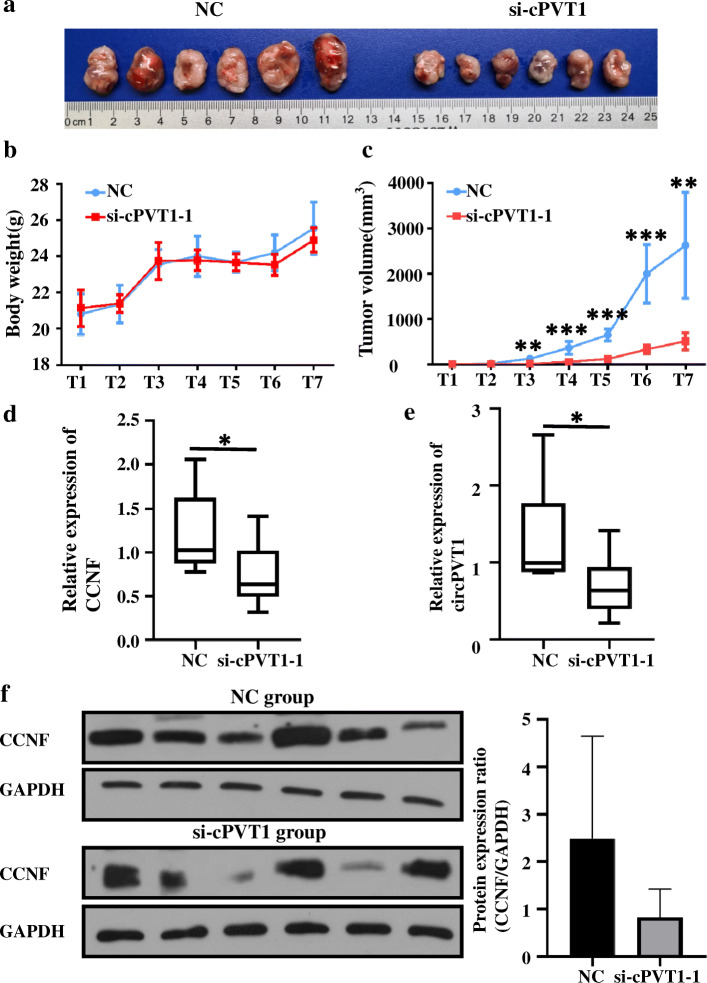
CircPVT1 suppressed tumor formation of xenograft in nude mice. **a** Representation picture of tumor formation of xenograft in nude mice (*n* = 6); **b** Body weight of mice which were measured every week; **c** Tumor volume of mice which were measured every week; **d** The expression level of CCNF was detected by qRT-PCR; **e** The expression level of circPVT1 was detected by qRT-PCR; **f** Proteins were extracted from the tumors and the protein expression of CCNF was measured using western blot. Data are shown as mean ± SD, *P < 0.05; **P < 0.01; ***P < 0.001

To verify the above findings, H520 cells were subcutaneously injected into nude mice. As shown in Fig. 7a, the development of tumors incubated from si-circPVT1 stably transfected cells was significantly slower than those tumors incubated from si-NC-transfected cells. Three weeks after cell inoculation, the average tumor volume, not the body weight, in the si-circPVT1 group was considerably lower compared to the NC group (Fig. 7b, c). Besides, through qRT-qPCR and western blot analysis, the expression contents of circPVT1 (Fig. 7e) and CCNF (Fig. 7d.f) were reduced in the tumors from the si-circPVT1 group.

## Discussion

In recent years, numerous circRNAs have been documented in tissues and cell lines. Based on cell/tissue-distinct and development stage-distinct expression and molecular structure of the circRNAs, they regulate various biological processes. Besides, they are potential diagnostic biomarkers or treatment targets for cancer, relative to linear transcripts [9]. Reports indicate that a few circRNAs act as oncogenes or tumor repressors in colon cancer [10], gastric cancer [11], breast cancer [12], liver cancer [13] and other types of cancer. Nonetheless, so far, only a few circRNAs have been completely characterized and the biological role of most of the circRNAs remains largely unclear.

Drugs targeting the ALK receptor tyrosine kinase (ALK), EGFR mutations, or ROS1 rearrangements have improved the outcomes of LUAD patients. This highlights the significance of molecular, genomic profiling and the subtyping of lung tumors [14–16]. However, in contrast with LUAD, only a few drug-gable genomic changes have been documented for LUSC, hence dismal LUSC patient outcomes. Therefore, understanding the molecular mechanisms underlying the development of LUSC might reveal the identification of novel therapeutic approaches. We utilized RNA-seq to acquire the expression patterns of circRNA and mRNA in LUSC tissues. Then, we uncovered circPVT1, which was upregulated in LUSC tissues and serum, and whose expression correlated with clinical-pathological data.

Previous studies have documented the role of circPVT1 in the progression of cancer and other diseases. For instance, Chen et al. reported that circPVT1 improves cell proliferation and acts as a prognostic index in gastric cancer [17]. Verduci et al. documented an oncogenic function of circPVT1 in head and neck squamous cell carcinoma through a mutant p53/YAP/TEAD transcription-competent complex [18]. Besides, Panda et al. opined that circPVT1 is senescence linked circular RNA, which is elevated in multiplying cells but diminished in senescent cells and sequesters let-7 to facilitate proliferation [19]. Elsewhere, Li et al discovered that transcription factor c-Fos regulate the expression of circPVT1, which mediates the progression of NSCLC. The NSCLC progression is achieved through the modulation of the E2F2 signaling cascade by binding to a miR-125b [20]. On the other hand, Qin et al revealed that circPVT1 acts as a competing endogenous RNA of miR-497 and indirectly modulates the expression of Bcl-2 [21]. While LUAD and LUSC are distinct in their histological, molecular, and clinical presentation, all the published experiments were performed in adenocarcinoma cells. We hypothesized that circPVT1 might be acting via different mechanisms in squamous cell carcinoma. In line with previous reports, our study reveals that circPVT1 serves as an oncogene in LUSC by different mechanisms unlike in LUAD.

The oncogenic influence of HuR was primarily attributed to its docking and stabilization of cancer-correlated mRNAs in the cytoplasm. Yang et al. discovered that circ-HuR downregulates the expression of HuR in gastric cancer [22]. CircPABPN1 blocks HuR binding to ATG16L1 mRNA and lowers ATG16L1 production [23]. Lan et al. established that the downregulation of lncOCC-1 triggers an escalation of HuR in Caco-2 cells [24]. Abdelmohsen et al. reported that masse circRNAs docks HuR in HeLa cells, CircPABPN1 being the most predominant. Further investigations illustrated that HuR did not impact CircPABPN1 richness, however, high contents of CircPABPN1 repressed HuR docking to PABPN1 mRNA [25]. Previous studies revealed that ncRNA (circRNA or lncRNA) regulates the expression of HuR. However, no data was demonstrating the effect of HuR on ncRNA abundance. For the first time, this study showed that HuR regulates the level of circPVT1, but the specific mechanism needs further investigations.

To get a profound understanding of the function of circPVT1 in LUSC, circRIP, RNA-seq, and bioinformatics were conducted to establish the downstream effector pathway after circPVT1 induction. Dual-luciferase reporter proved that circPVT1 could directly interact with miR-30d and miR-30e. Previous reports have revealed that miR-30d was a tumor suppressor in prostate cancer, ovarian cancer among other types of cancer [26, 27]. Besides, it is a potential non-invasive diagnostic biomarker in cervical cancer, rectal cancer, and NSCLC [28–30]. In NSCLC, miR-30d represses tumor cell proliferation and motility by directly targeting CCNE2 and NFIB [31, 32]. On the other hand, miR-30e acted as an oncogene in hepatocellular carcinoma, breast cancer among other types of cancer [33, 34]. In NSCLC, it inhibited cell proliferation and infiltration by directly targeting SOX9, PTPN1, and USP22-mediated Sirt1/JAK/STAT3 signaling [8, 35, 36]. Additionally, miR-30e was presented to be a potential non-invasive diagnostic biomarker for NSCLC [30]. Our data illustrated that circPVT1 acts as an oncogene by sponging miR-30d and miR-30e in LUSC. Further, we highlighted the value of the cross-talk between circPVT1 and miR-30d or miR-30e in tumorigenesis and development of LUSC.

Reports hypothesize that circRNA acts as a ceRNA and modulates the expression of a miRNA target gene. Through bioinformatics, we found that miR-30d and miR-30e might play a vital role by sponging key genes. Based on qRT-PCR and dual-luciferase reporter assays, we discovered that miR-30d or miR-30e directly target the 3′-untranslated regions of CCNF. Besides, down-expression of miR-30d or miR-30e caused the up-regulation of CCNF at mRNA and protein levels. CCNF is the founding member of the F-box family of proteins, the substrate recognition subunits of Skp1-Cul1-F-box protein (SCF) ubiquitin ligase complexes. CCNF degradation permits rapid aggregation of RRM2 in response to genotoxic stress [37]. A secondary response that involves p53R2, a paralog of RRM2, whose transcription was induced by p53 [38]. P53R2 is likely defective in approximately 50% of human cancers bearing p53 mutations, making CCNF closely related to the development of cancer. In malignant germ cell tumors, CCNF participated in the LIN28/let-7 pathway as an oncogene [39]. Acute myeloid leukemia patients with long-term proliferation demonstrated altered expression of CCNF [40]. Using the TCGA dataset, CCNF was significantly up modulated in LUSC tissues. So far, the role of CNNF in NSCLS remain unreported. As such, we conducted in vitro experiments to confirm the role of CCNF in the proliferation of LUSC cells, which was following a previous study on the role of CCNF in cancers and the CCNF expression in LUSC in TCGA. To confirm the interaction between circPVT1 and CCNF, down-regulation of circPVT1 downregulated CCNF at both the mRNA and protein expression levels. Moreover, these impacts could be partly alleviated by miR-30d or miR-30e inhibitors. The data support our hypothesis that circPVT1 acts as a ceRNA to improve CCNF-mediated proliferation by sponging miR-30d and miR-30e in LUSC.

## Conclusion

In conclusion, our findings revealed that elevated expression of circPVT1 is a potential independent prognostic marker for LUSC. Moreover, we demonstrated that HuR regulates the level of circPVT1 and circPVT1 might sponge miR-30d and miR-30e to modulate CCNF expression, thereby causing tumorigenesis and development of LUSC. We further inferred that circPVT1 is a potential diagnostic marker and a potential therapy target for LUSC. Finally, the regulatory network involving HuR/circPVT1/miR-30d and miR-30e/CCNF cascade potentially provides a profound understanding of the mechanism of pathogenesis and progression of LUSC.

## Supplementary Information

**Additional file 1: Table S1.** Sequences of primers used in this study. **Table S2.** Sequences of siRNAs used in this study.

**Additional file 2: Figure S1.** A luciferase reporter assay was used to detect the luciferase activity of LUC-cPVT1 in T293 cells transfected with miRNA mimics to identify miRNAs that bind to the circPVT1 sequence. Data are shown as mean ± SD, ***P < 0.001.

**Additional file 3: Figure S2.** qRT-PCR analysis of circPVT1 expression in H520 cells after treatment with miR-30d minics or miR-30e minics or miR-30d inhibitors or miR-30e inhibitors.

**Additional file 4: Figure S3.** qRT-PCR analysis of mRNA expression in H520 cells after treatment with si-cPVT1–1 and si-cPVT1–2.

**Additional file 5: Figure S4.** Assessment of proliferation in H520 cells transfected with NC, si-CCNF-1, siCCNF-2, si-CCNF-3 by CCK-8 assay. *P < 0.05;**P < 0.01; ***P < 0.001.

## Data Availability

All data generated or analyzed during this study are included in this published article and its additional information files.

## Abbreviations

ceRNA: Competing endogenous RNA
CircRNA: Circular RNA
LUSC: Lung squamous cell carcinoma
qRT-PCR: Real-time quantitative polymerase chain reaction
CCNF: Cyclin F
SCLC: Small cell lung cancer
LUAD: Lung adenocarcinoma
NSCLC: Non-small cell lung cancer
MREs: MicroRNA response elements
IHC: Immunohistochemistry
miRNA: MicroRNA
RIP: RNA immunoprecipitation
PPI: Protein-protein interaction
RNA-seq: RNA sequencing
TCGA: The Cancer Genome Atlas
DEPDC1B: DEP domain containing protein 1B
ALK: ALK receptor tyrosine kinase
SCF: Skp1-Cul1-F-box protein
