# Supplementary material for: CircPVT1 promotes proliferation of lung squamous cell carcinoma by binding to miR-30d/e
Source: J Exp Clin Cancer Res. 2021 Jun 10;40:193. doi: 10.1186/s13046-021-01976-w (PMC8194141; doi:10.1186/s13046-021-01976-w)
Supplement: Supplementary file 1 — Additional file 1: Table S1. Sequences of primers used in this study. Table S2. Sequences of siRNAs used in this study. [file 13046_2021_1976_MOESM1_ESM.docx]

**Table S1. Sequences of primers used in this study.**

| Gene | Primer sequences |
| --- | --- |
| circPVT1 | F: 5’-ATGGAATGTAAGACCCCGACTC-3’ |
|  | R: 5’-GACCACTGAAGATCACTGTAAATCC-3’ |
| PVT1 | F: 5’-AGCTGGCTGAGAGGGTTGAG-3’  R: 5’-TCGGGGTCTTACATTCCATAGGG-3’ |
| HuR | F: 5’- TGTTCTCTCGGTTTGGGCGGAT-3’  R: 5’- TCTTCTGCCTCCGACCGTTTGT-3’ |
| CCNF | F: 5’-AGGACAAGCGCTATGGAGAA-3’ |
|  | R: 5’-TCTGTCTTCCTGGAGGCTGT-3’ |
| DEPDC1B | F: 5’-TGGCAGTTGAAGCATTTCAG-3’  R: 5’-GATGCAACGGGAAAATGTCT-3’ |
| GAPDH | F: 5’-GAAGGTGAAGGTCGGAGTC-3’ |
|  | R: 5’-GAAGATGGTGATGGGATTTC-3’ |

**Table S2. Sequences of siRNAs used in this study.**

| Definition | sequences |
| --- | --- |
| si-cPVT1-1 | GCUUGAGCCUGAUCUUUUTT |
| si-cPVT2-2 | CUUGAGGCCUGAUCUUUUG |
| si-NC | TTCTCCGAACGTGTCACGT |
| si-HuR-1 | GGGCTATATCCATCGAAATTT |
| si-HuR-2 | ACGACAATGGAAGCGGATATA |
| si-CCNF-1  si-CCNF-2  si-CCNF-3 | AGGAAGAGGUGCUGAGCUATT  UGAAGGACCUGGUGGACAATT  CCUACAAAGCGAAGAAUAATT |

CircPVT1 Sequence:

GCCTGATCTTTTGGCCAGAAGGAGATTAAAAAGATGCCCCTCAAGATGGCTGTGCCTGTCAGCTGCATGGAGCTTCGTTCAAGTATTTTCTGAGCCTGATGGATTTACAGTGATCTTCAGTGGTCTGGGGAATAACGCTGGTGGAACCATGCACTGGAATGACACACGCCCGGCACATTTCAGGATACTAAAAGTGGTTTTAAGGGAGGCTGTGGCTGAATGCCTCATGGATTCTTACAGCTTGGATGTCCATGGGGGACGAAGGACTGCAGCTGGCTGAGAGGGTTGAGATCTCTGTTTACTTAGATCTCTGCCAACTTCCTTTGGGTCTCCCTATGGAATGTAAGACCCCGACTCTTCCTGGTGAAGCATCTGATGCACGTTCCATCCGGCGCTCAGCTGGGCTTGAG
