# Supplementary figures and images for: CircPVT1 promotes proliferation of lung squamous cell carcinoma by binding to miR-30d/e
Source: J Exp Clin Cancer Res. 2021 Jun 10;40:193. doi: 10.1186/s13046-021-01976-w (PMC8194141; doi:10.1186/s13046-021-01976-w)

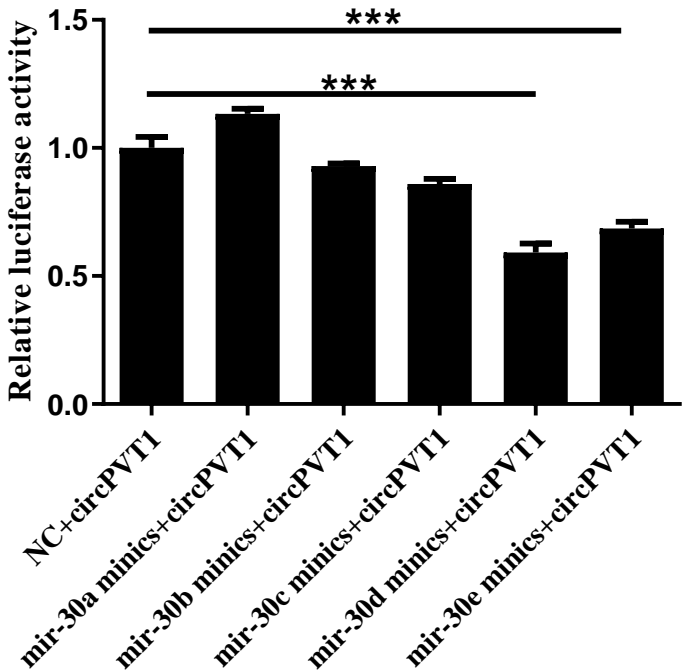

Supplement: Supplementary file 2 — Additional file 2: Figure S1. A luciferase reporter assay was used to detect the luciferase activity of LUC-cPVT1 in T293 cells transfected with miRNA mimics to identify miRNAs that bind to the circPVT1 sequence. Data are shown as mean ± SD, ***P < 0.001. [file 13046_2021_1976_MOESM2_ESM.pdf]

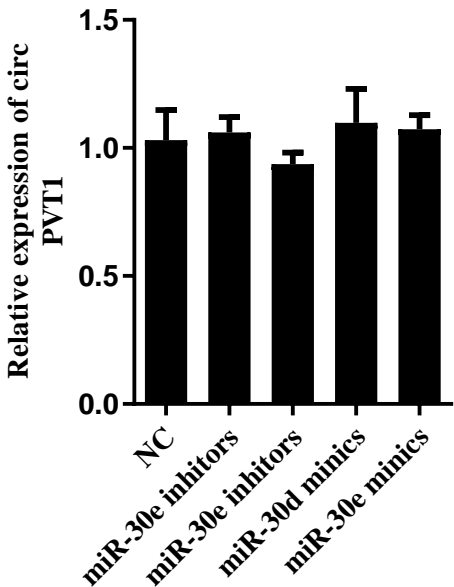

Supplement: Supplementary file 3 — Additional file 3: Figure S2. qRT-PCR analysis of circPVT1 expression in H520 cells after treatment with miR-30d minics or miR-30e minics or miR-30d inhibitors or miR-30e inhibitors. [file 13046_2021_1976_MOESM3_ESM.pdf]

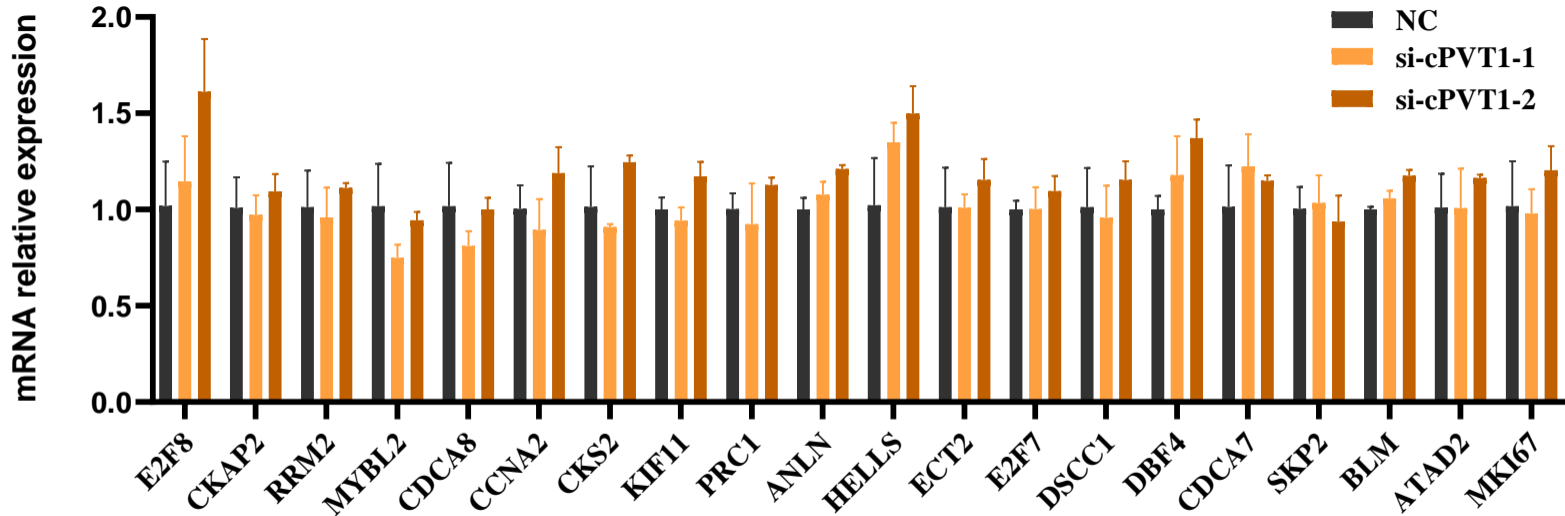

Supplement: Supplementary file 4 — Additional file 4: Figure S3. qRT-PCR analysis of mRNA expression in H520 cells after treatment with si-cPVT1–1 and si-cPVT1–2. [file 13046_2021_1976_MOESM4_ESM.pdf]

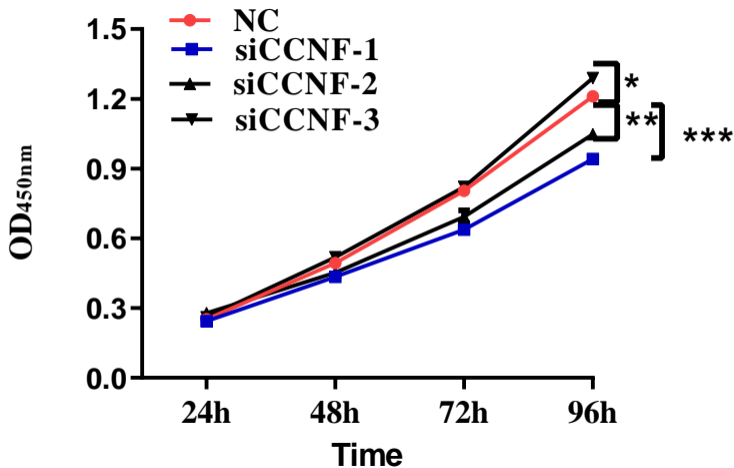

Supplement: Supplementary file 5 — Additional file 5: Figure S4. Assessment of proliferation in H520 cells transfected with NC, si-CCNF-1, siCCNF-2, si-CCNF-3 by CCK-8 assay. *P < 0.05;**P < 0.01; ***P < 0.001. [file 13046_2021_1976_MOESM5_ESM.pdf]
